# Synthesis of spiropyrans: H-abstractions in 3-cycloalkenyloxybenzopyrans

**DOI:** 10.1186/1860-5397-3-14

**Published:** 2007-03-21

**Authors:** Satish C Gupta, Mandeep Thakur, Somesh Sharma, Urmila Berar, Surinder Berar, Ramesh C Kamboj

**Affiliations:** 1 Department of Chemistry, Kurukshetra University, Kurukshetra-136119, India

## Abstract

A photochemical route for the synthesis of some benzopyronospiropyrans from 2-furyl-3-cycloalkenyloxybenzopyrones involving H-abstraction is reported. How a methyl group on the furyl ring affects the product formation is also investigated.

## 1. Background

Spiropyrans exhibit photochromism due to the photoequilibrium with their open chain analogues merocyanins – a property that makes them a material of choice in digital storage technology. [1–5] Some spiropyrans have been found to have antiphlogistic, spasmolytic, antidiabetic and antifeedant activities amongst others. [6–10] The methods available for the synthesis of spiropyrans include enamine eliminations, reductive/thermal cyclisations and [4+2] cycloadditions. [11–16] These methods being specific in nature for a particular spirocyclic compound, offer limited synthetic utility. In the past, we have investigated the use of photochemical methods for the synthesis of organic molecules like vinyl ethers, [17] pyranopyrones [18–19] etc. and in continuation of that work we published a preliminary report on photochemical synthesis of spiropyrans. [20] To extend and examine the scope of this methodology, in this communication, we report the synthesis of spiropyrans bearing dihydrofuryls and acylcyclopropanes.

## 2. Results

The substrates **1** and **2** required for this study were obtained through the alkylation of 6-chloro-2-(2'-furyl)-3-hydroxy-4-oxo-4*H*-1-benzopyrans [21]**3** (R = H) and **4** (R = CH_3_) with an appropriate bromocycloalkene (R' Br).

**Scheme 1:**
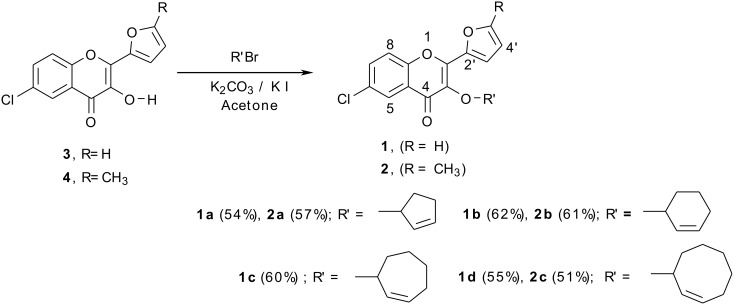
Synthesis of 3-Cycloalkenyloxybenzopyrans.

The irradiation of **1c** in methanol with pyrex filtered light from a 125W Hg lamp produced two products **5c** and **6c** along with some **3**.

**Scheme 2:**
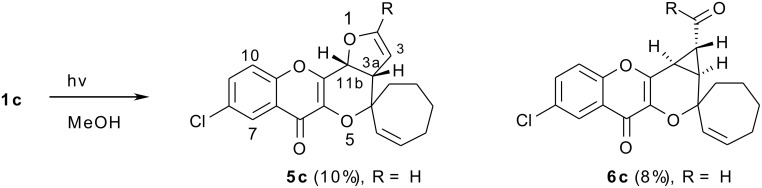
Photolysis of 6-chloro-3-(1"-cyclohept-2"-enyloxy)-2-(2'-furyl)-4-oxo-4*H*-1-benzopyran.

## 3. Discussion

The structures of the photoproducts **5c** and **6c** were consistent with their spectral parameters [see Supporting Information File 1]; both the spiropyrans exhibited the presence of ions at m/z 155 and m/z 202 values in their mass spectra; these ions could very well be the result of rDA fragmentation of **5c** and **6c**. In the mass spectrum of **6c**, the base peak was at m/z 327 (M^+^ -CHO).

The other chromones **1a, 1b** and **1d** on photoirradiation rearranged to spirocyclic pyranopyrones **5a, 5b, 5d** and **6a, 6b, 6d**. The photoirradiation of **2a, 2b** and **2c** yielded **7a, 7b, 7c** only and no photoproducts similar to **5** (R = CH_3_) could be isolated although the ^1^H NMR of the crude photolysate of **2b** did show the presence of **5b** (R = CH_3_) in small proportions.

**Scheme 3:**
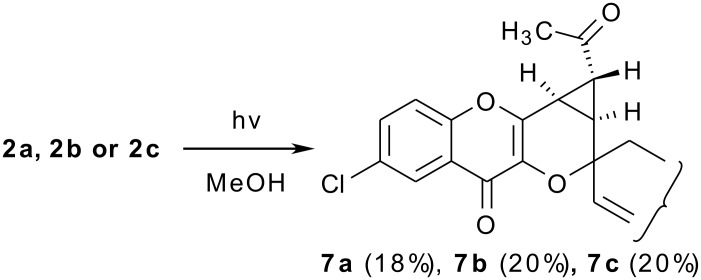
Photolysis of 6-chloro-3-(1"-cycloalk-2"-enyloxy)-2-[2'-(5-methylfuryl)]-4-oxo-4*H*-1-benzopyrans.

These photoconversions of the cycloalkenyloxy chromones (**1a**, **1b**, **1c**, **1d**, **2a**, **2b** and **2c**) to pyronospiropyrans can be visualized as having occurred through an initial abstraction of the *O*-methine proton of cycloalkenyl group by the excited carbonyl of the pyrone moiety to produce 1,4-biradical **8**. The photoproduct **5** (**a**, **b**, **c**, **d**), is then formed through bond formation between the alkoxy radical and furan (**8**), followed by 1,5-H migration in **8a**. [21]

**Scheme 4:**
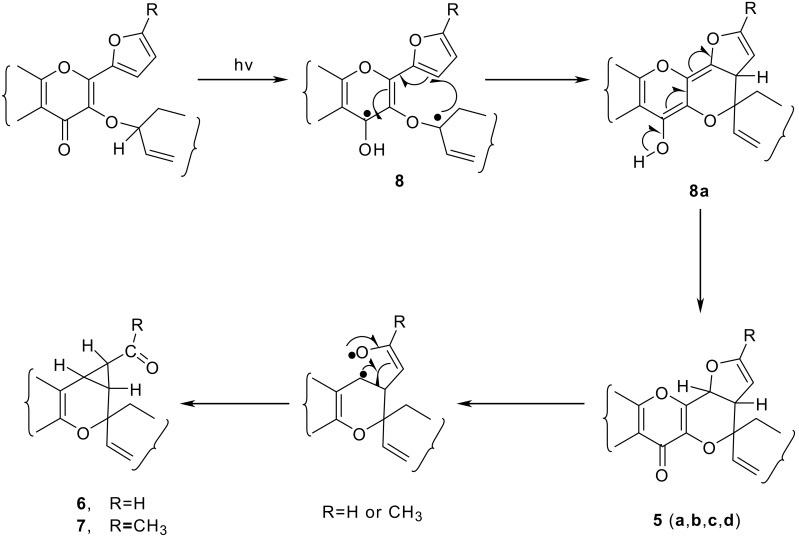
Mechanism of product formation from 3-Cycloalkenyloxybenzopyrans by photolysis.

The spiropyrans **6** (R = H) bearing cyclopropanes are the secondary photoproducts formed as a result of the further reorganization of **5** (R = H), through a ring contraction-ring expansion mechanism. [22–23] The formation of acetylcyclopropane compound **7** (R = CH_3_) from **2** (R = CH_3_) can be explained likewise. However, it is pertinent to mention here that when a large amount of **2b** was photolysed and the photolysate was carefully chromatographed, a very small amount (<3%) of photoproduct **5** (R = CH_3_) could be isolated (vide experimental). The reason for such behaviour of **2** (R = CH_3_) could be that the presence of -CH_3_ group on dihydrofuryl moiety in the photoproduct **5** (R = CH_3_) makes the C-O bond easier to cleave. Thus as soon as **5** (R = CH_3_) is formed, it rearranges to **7** (R = CH_3_) or in other words the conversion of **5** (R = CH_3_) into **7** (R = CH_3_) is much faster than that of the formation of **5** (R = CH_3_) from **2** (R = CH_3_).

Regarding the stereochemical disposition of H-11b and H-3a in **5**, both of them are *cis* placed (*J* = 9.6–9.9 Hz, Ø = 19.2°); in photoproducts bearing cyclopropanes [21]**6** (R = H) or **7** (R = CH_3_), H-1 is *trans* to H-2 and H-3 (*J*_1,2_ = 4.8 Hz, *J*_1,3_ = 3.6 Hz) and both H-2 and H-3 are *cis* (*J*_2,3_ = 9.0 Hz).

## 4. Conclusion

In conclusion this photochemical method can be of utility for synthesizing a variety of pyronospiropyrans bearing both dihydrofuryls and acylcyclopropanes. The substitution on the furan ring makes the primary photoproducts more amenable to cleavage.

## Supporting Information

File 1 Experimental Data. The data provided represents the yield, melting point, IR, ^1^H NMR and elemental analysis of the compounds.
