# Supplementary material for: Synthesis of spiropyrans: H-abstractions in 3-cycloalkenyloxybenzopyrans
Source: Beilstein J Org Chem. 2007 Mar 21;3:14. doi: 10.1186/1860-5397-3-14 (PMC1847525; doi:10.1186/1860-5397-3-14)
Supplement: File 1 — Experimental Data. The data provided represents the yield, melting point, IR, 1H NMR and elemental analysis of the compounds. [file Beilstein_J_Org_Chem-03-14-s001.doc]

**Experimental**

**General**

IR spectra were recorded on a Buck Scientific 500 spectrophotometer using KBr pellets and UV spectra on U-2000 Hitachi-Spectrophotometer. ^1^H NMR spectra were recorded on a 300 MHz Bruker spectrometer using TMS as internal standard. Melting points were determined in open capillaries and are uncorrected.

**General method for the synthesis of 3-cyclopentenyloxy-6-chloro-2-(2'-furyl)-4-oxo-4*H*-1-benzopyran 1a.**

The 3-hydroxychromone, **3** (2.62 g, 0.01mol), 3-cyclopentenyl bromide (1.67 g, 0.012mol), dry K_2_CO_3_ (1.0 g) and tetrabutylammonium iodide (200 mg) were refluxed in acetone (25 ml) for 4h. The reaction mixture was filtered, evaporated and the residue was chromatographed to obtain **1a.**

The other compounds **1b, 1c** and **1d** were obtained by using this procedure and the compounds **2a-2c** were obtained starting from **4**.

**Photolysis of 3-cycloalkenyloxy-6-chloro-2-(2'-furyl)-4-oxo-4*H*-1-benzopyran, 1a-1d.**

A general procedure: A deoxygenated methanol solution of the compound **1a** (200 mg, 0.0006 mol) was photolysed with light from a 125 W mercury lamp, in a pyrex reactor under nitrogen atmosphere 35min. The photolysate was chromatographed over a column of silica-gel (100-200 mesh) to yield **1a** (30%, mmp), **5a and 6a.**

**Photolysis of 3-cycloalkenyloxy-6-chloro-2-(5**'**-methyl-2'-furyl)-4-oxo-4*H*-1-benzopyran 2a-2c.**

A deoxygenated methanolic solution of the compound **2a** (200 mg, 0.0006 mol) was photolysed with light from a 125 W mercury lamp, in a pyrex reactor under similar condition as used in **5.1.3** to yield **2a** (35%, mmp) and **7a**(R = CH_3_).

Experimental Data

Compound **1a** (R = H)**.** Yield 54%, white solid; mp 78-80 ºC; *λ*_max_ THF(nm) 257, 348 *ν*_max_(cm^-1^) 1639 (C=O), 1602 (C=C); δ_H_ (CDCl_3_) 8.20 (1H, d, *J*_m_=2.4 Hz, H-5), 7.72 (1H, d, *J*_5',4'_=1.7 Hz, H-5'), 7.64 (1H, dd, *J*_m,o_ 2.4 Hz, 9.0 Hz, H-7), 7.55 (1H, d, *J*_m_=9.0 Hz, H-8), 7.42 (1H, d, *J*_3',4'_ 3.6 Hz, H-3'), 6.60 (1H, dd, *J*_4',5'_=1.7 Hz, *J*_4',3'_=3.6 Hz, H-4'), 5.88-5.81 (2H, m, H- 2",3"), 5.55-6.48 (1H, m, H-1"), 2.53-1.97 (4H, m, H-4",5"). MS (EI): *m/z* 328 (M^+^, 100%). Anal. Calcd. for C_18_H_13_ClO_4_: C, 65.76; H, 3.99. Found: C, 65.79; H, 3.95.

Compound **1b**(R = H)**.** Yield 62%, white solid; mp 115-118 ºC; *λ*_max_ THF(nm) 260, 353 *ν*_max_(cm^-1^) 1638 (C=O), 1602 (C=C); δ_H_ (CDCl_3_) 8.16 (1H, d, *J*_m_=2.4 Hz, H-5), 7.67 (1H, d, *J*_5',4'_=1.7 Hz, H-5'), 7.60 (1H, dd, *J*_m,o_ 2.4 Hz, 9.0 Hz, H-7), 7.50 (1H, d, *J*_m_=9.0 Hz, H-8), 7.42 (1H, d, *J*_3',4'_ 3.6 Hz, H-3'), 6.60 (1H, dd, *J*_4',5'_=1.7 Hz, *J*_4',3'_=3.6 Hz, H-4'), 5.90-5.82 (2H, m, H- 2",3"), 5.45-5.42 (1H, m, H-1"), 2.12-1.72 (6H, m, H-4",5",6"). MS (EI): *m/z* 342 (M^+^, 100%). Anal. Calcd. for C_19_H_15_ClO_4_: C, 66.58 ; H, 4.41. Found: C, 66.55; H, 4.39.

Compound **1c**(R = H)**.** Yield 60%, white solid; mp 85-87 ºC; *λ*_max_ THF(nm) 259, 348; *ν*_max_(cm^-1^) 1642(C=O), 1602 (C=C); δ_H_ (CDCl_3_) 8.20 (1H, d, *J*_m_=2.4 Hz, H-5), 7.73 (1H, d, *J*_5',4'_=1.7 Hz, H-5'), 7.62 (1H, dd, *J*_m,o_ 2.4 Hz, 9.0 Hz, H-7), 7.55 (1H, d, *J*_m_=9.0 Hz, H-8), 7.45 (1H, d, *J*_3',4'_ 3.6 Hz, H-3'), 6.65 (1H, dd, *J*_4',5'_=1.7 Hz, *J*_4',3'_=3.6 Hz, H-4'), 5.95-5.79 (2H, m, H- 2",3"), 5.67-5.62 (1H, m, H-1"), 2.21-1.61 (8H, m, H-4",5",6",7"). MS (EI): *m/z* 356 (M^+^, 100%). Anal. Calcd. for C_20_H_17_ClO_4_: C, 67.32; H, 4.80. Found: C, 67.35; H, 4.78.

Compound **1d**(R = H)**.** Yield 55%, white solid; mp 125-126 ºC; *λ*_max_ THF(nm) 256, 325 *ν*_max_(cm^-1^) 1639 (C=O), 1606 (C=C); δ_H_ (CDCl_3_) 8.17 (1H, d, *J*_m_=2.4 Hz, H-5), 7.70 (1H, d, *J*_5',4'_=1.7 Hz, H-5'), 7.58 (1H, dd, *J*_m,o_ 2.4 Hz, 9.0 Hz, H-7), 7.55 (1H, d, *J*_m_=9.0 Hz, H-8), 7.45 (1H, d, *J*_3',4'_ 3.6 Hz, H-3'), 6.65 (1H, dd, *J*_4',5'_=1.7 Hz, *J*_4',3'_=3.2 Hz, H-4'), 5.96-5.92 (1H, m, H-3"), 5.62-5.45 (2H, m, H-2",1"), 2.25-2.18 (2H, m, H-4"), 2.01-1.65 (8H, m, H-5",6",7",8"). MS (EI): *m/z* 370 (M^+^, 100%). Anal. Calcd. for C_21_H_19_ClO_4_ : C, 68.02; H, 5.16. Found: C, 68.05; H, 5.14.

Compound **2a**(R = CH_3_)**.** Yield 57%, white solid; mp 106-107 ^o^C; *ν*_max_ (cm^-1^) 1640 (C=O), 1603 (C=C); δ_H_ (CDCl_3_) 8.20 (1H, d, *J*_m_=2.4 Hz, H-5), 7.62 (1H,dd, *J*_m,o_=2.4 Hz, 9.0 Hz, H-7), 7.54 (1H, d, *J*_o_=9.0 Hz, H-8), 7.41 (1H, d, *J*_3',4'_=3.6 Hz, H-3'), 6.23 (1H, dd, *J*_4',3'_=3.6 Hz, *J*_allyl_=9.0 Hz, H-4'), 5.92-5.83 (2H, m, H-2",3"), 5.38-5.30 (1H, m, H-1"), 2.41 (3H, s, –CH_3_), 2.19-1.87 (4H, m, H-4",5"). MS (EI): *m/z* 342 (M^+^, 100%). Anal. Calcd. for C_19_H_15_ClO_4_: C, 66.58 ; H, 4.41. Found: C, 66.60; H, 4.39.

Compound **2b**(R = CH_3_)**.** Yield (61%), white solid; mp 114-116 ^o^C; *ν*_max_ (cm^-1^) 1641(C=O) 1602 (C=C); δ_H_ (CDCl_3_) 8.19 (1H, d, *J*_m_=2.4 Hz, H-5), 7.60 (1H, dd, *J*_m,o_=2.4 Hz, 9.0 Hz, H-7), 7.49 (1H, d, *J*_o_=9.0 Hz, H-8), 7.39 (1H, d, *J*_3',4'_=3.6 Hz, H-3'), 6.18 (1H, dd, *J*_4',3'_=3.6, Hz, *J*_allyl_=9.0 Hz, H-4'), 5.98-5.94 (2H, m, H-2",3"), 5.50-5.42 (1H, m, H-1"), 2.44 (3H, s, –CH_3_), 2.22-1.60 (6H, m, H-4",5",6"). MS (EI): *m/z* 356 (M^+^, 100%). Anal. Calcd. for C_20_H_17_ClO_4_: C, 67.32; H, 4.80. Found: C, 67.28; H, 4.82.

Compound **2c**(R = CH_3_)**.** Yield 51%, white solid; mp 126-128 ^o^C; *ν*_max_ (cm^-1^) 1640 (C=O), 1603 (C=C); δ_H_ (CDCl_3_) 8.23 (1H, d, *J*_m_=2.4 Hz, H-5), 7.65 (1H,dd, *J*_m,o_=2.4 Hz, 9.0 Hz, H-7), 7.53 (1H, d, *J*_o_=9.0 Hz, H-8), 7.40 (1H, d, *J*_3',4'_=3.6 Hz, H-3'), 6.25 (1H, dd, *J*_4',3'_=3.6 Hz, *J*_allyl_=9.0 Hz, H-4'), 5.98-5.90 (1H, m, H-3"), 5.55-5.46 (2H, m, H-1",2"), 2.48 (3H, s, -CH_3_), 2.28-2.19 (2H, m, H-4'), 2.05-1.57 (8H, m, H-5",6",7",8"). MS (EI): *m/z* 384 (M^+^, 100%). Anal. Calcd. for C_22_H_21_ClO_4_: C, 68.66; H, 5.50. Found: C, 68.3; H, 5.47.

Compound **5a**(R = H)**.** Yield 10%, white solid; mp 173-175 ^o^C; *ν*_max_ (cm^-1^) 1605 (C=C), 1652 (C=O); δ_H_ (CDCl_3_) 8.20 (1H, d, *J*_m_=2.4 Hz, H-7), 7.60 (1H, dd, *J*_m,o_=2.4 Hz, 9.0 Hz, H-9), 7.47 (1H, d, *J*_o_=9.0 Hz, H-10), 6.54 (1H, d, *J*_2,3_=2.7 Hz, H-2), 6.15-6.12 (1H, m, H-3'), 5.81-5.79 (1H, m, H-2'), 5.15 (1H, d, *J*_11b,3a_=9.6 Hz, H-11b), 5.07-5.05 (1H, m, H-3), 3.51-3.47 (1H, d, *J*_3a ,11b_=9.6 Hz, H-3a), 2.23-1.90 (4H, m, H-4',5'). MS (EI): *m/z* 328 (M^+^, 100%). Anal. Calcd. for C_18_H_13_ClO_4_: C, 65.76; H, 3.99. Found: C, 65.73; H, 3.97.

Compound **6a** (R = H)**.** Yield 3%, white solid; mp 185-186 ^o^C; *ν*_max_ (cm^-1^) 1695 (C=O), 1637 (C=O), 1605 (C=C); δ_H_ (CDCl_3_) 9.68 (1H, d, *J=*2.4 Hz, -CHO), 8.18 (1H, d, *J*_m_=2.4 Hz, H-7), 7.54 (1H, dd, *J*_m,o_=2.4 Hz, 9.0 Hz, H-9), 7.38 (1H, d, *J*_o_=9.0 Hz, H-10), 5.96-5.91 (1H, m, H-3'), 5.64-5.59 (1H, m, H-2'), 2.90 (1H, dd, *J*_1,3_=3.6 Hz, *J*_1,2_=4.8 Hz, H-1), 2.74 (1H, dd, *J*_3,1_=3.6 Hz, *J*_3,2_=9.0 Hz, H-3), 2.56 (1H, dd, *J*_2,1_=4.8 Hz, *J*_2,3_=9.0 Hz, H-2), 2.17-1.99 (4H, m, H-4',5'). MS (EI): *m/z* 328 (M^+^, 100%). Anal. Calcd. for C_18_H_13_ClO_4_: C, 65.76; H, 3.99. Found: C, 65.74; H, 3.96.

Compound **5b**(R = H)**.** Yield 26%, white solid; mp 178-180 ^o^C; *ν*_max_ (cm^-1^) 1644 (C=O), 1603 (C=C); δ_H_ (CDCl_3_) 8.18 (1H, d, *J*_m_=2.4 Hz, H-7), 7.55 (1H, dd, *J*_m,o_=2.4 Hz, 9.0 Hz, H-9), 7.40 (1H, d, *J*_o_=9.0 Hz, H-10), 6.50 (1H, d, *J*_2,3_=2.7 Hz, H-2), 6.10-6.02 (1H, m, H-3'), 5.70 (1H, d, *J*_2',3'_=10.0 Hz, H-2'), 5.35 (1H, d, *J*_11b,3a_=9.6 Hz, H-11b), 5.10-5.07 (1H, m, H-3), 3.45 (1H, d, *J*_3a ,11b_=9.6 Hz, H-3a), 2.22-1.68 (6H, m, H-4',5',6'). MS (EI): *m/z* 356 (M^+^, 100%). Anal. Calcd. for C_20_H_17_ClO_4_: C, 67.32; H, 4.80. Found: C, 67.29; H, 4.79.

Compound **6b**(R = H)**.** Yield 15%, white solid; mp 192-195 ^o^C; *ν*_max_ (cm^-1^) 1696 (C=O), 1642 (C=O), 1605 (C=C); δ_H_ (CDCl_3_) 9.70 (1H, d, *J=*2.4 Hz, -CHO), 8.20 (1H, d, *J*_m_=2.4 Hz, H-7), 7.56 (1H, dd, *J*_m,o_=2.4 Hz, 9.0 Hz, H-9), 7.40 (1H, d, *J*_o_=9.0 Hz, H-10), 5.98-5.93 (1H, m, H-3'), 5.62 (1H, d, *J*_2',3'_=10.0 Hz, H-2'), 2.92 (1H, dd, *J*_1,3_=3.6 Hz, *J*_1,2_=4.8 Hz, H-1), 2.76 (1H, dd, *J*_3,1_=3.6 Hz, *J*_3,2_=9.0 Hz, H-3), 2.58 (1H, dd, *J*_2,1_=4.8 Hz, *J*_2,3_=9.0 Hz, H-2), 2.24-1.73 (6H, m, H-4',5',6'). MS (EI): *m/z* 356 (M^+^, 100%). Anal. Calcd. for C_20_H_17_ClO_4_: C, 67.32; H, 4.80. Found: C, 7.29; H, 4.82.

Compound **5c**(R = H)**.** Yield 10%, white solid; mp 163-165 ^o^C; *ν*_max_ (cm^-1^) 1655 (C=O), 1605 (C=C); δ_H_ (CDCl_3_) 8.20 (1H, d, *J*_m_=2.4 Hz, H-7), 7.57 (1H, dd, *J*_m,o_=2.4 Hz, 9.0 Hz, H-9), 7.45 (1H, d, *J*_o_=9.0 Hz, H-10), 6.42 (1H, d, *J*_2,3_=2.7 Hz, H-2), 5.97-5.88 (1H, m, H-3'), 5.70 (1H, d, *J*_2',3'_=11.7 Hz, H-2'), 5.38 (1H, d, *J*_11b,3a_=9.9 Hz, H-11b), 5.10-5.07 (1H, m, H-3), 3.85 (1H, d, *J*_3a ,11b_=9.9 Hz, H-3a), 2.39-1.88 (8H, m, H-4',5',6',7'). MS (EI): *m/z* 356 (M^+^, 100%). Anal. Calcd. for C_20_H_17_ClO_4_: C, 67.32; H, 4.80. Found: C, 67.29; H, 4.82.

Compound **6c**(R = H)**.** Yield 8%, white solid; mp 168-169 ^o^C; *ν*_max_ (cm^-1^) 1695 (C=O), 1648 (C=O), 1610 (C=C); δ_H_ (CDCl_3_) 9.74 (1H, d, *J=*2.4 Hz, -CHO), 8.20 (1H, d, *J*_m_=2.4 Hz, H-7), 7.56 (1H, dd, *J*_m,o_=2.4 Hz, 9.0 Hz, H-9), 7.40 (1H, d, *J*_o_=9.0 Hz, H-10), 5.92-5.86 (1H, m, H-3'), 5.50 (1H, d, *J*_2',3'_=11.7 Hz, H-2'), 2.90 (1H, dd, *J*_1,3_=3.6 Hz, *J*_1,2_=4.8 Hz, H-1), 2.75 (1H, dd, *J*_3,1_=3.6 Hz, *J*_3,2_=9.0 Hz, H-3), 2.49 (1H, dd, *J*_2,1_=4.8 Hz, *J*_2,3_=9.0 Hz, H-2), 2.34-1.73 (8H, m, H-4',5',6',7'). MS (EI): *m/z* 356 (M^+^, 100%). Anal. Calcd. for C_20_H_17_ClO_4_: C, 67.32; H, 4.80. Found: C, 67.31; H, 4.78.

Compound **5d**(R = H)**.** Yield 12%, white solid; mp 174-176 ^o^C; *ν*_max_ (cm^-1^) 1646 (C=O), 1610 (C=O); δ_H_ (CDCl_3_) 8.13 (1H, d, *J*_m_ 2.4 Hz, H-7), 7.50 (1H, dd, *J*_m,o_=2.4 Hz, 9.0 Hz, H-9), 7.37 (1H, d, *J*_o_=9.0 Hz, H-10), 6.34 (1H, d, *J*_2,3_=2.7 Hz, H-2), 5.70-5.60 (1H, m, H-3'), 5.39 (1H, d, *J*_2',3'_=11.7 Hz, H-2'), 5.28 (1H, d, *J*_11b,3a_=9.9 Hz, H-11b), 5.05 (1H, t, *J* =2.7 Hz, H-3), 3.52 (1H, d{t}, *J*=9.9 Hz, 2.7 Hz, H-3a), 2.81-2.76 (1H, m, H-4'), 2.28 - 2.24 (1H, m, H-4'), 2.02-1.47 (8H, m, H-5',6',7',8'). MS (EI): *m/z* 384 (M^+^, 100%). Anal. Calcd. for C_22_H_21_ClO_4_: C, 68.66; H, 5.50. Found: C, 68.68; H, 5.48.

Compound **6d**(R = H)**.** Yield 3%, white solid; mp 168-171 ^o^C; *ν*_max_ (cm^-1^) 1692 (C=O), 1645 (C=O), 1617 (C=C); δ_H_ (CDCl_3_) 9.65 (1H, d, *J=*2.4 Hz, -CHO), 8.18 (1H, d, *J*_m_=2.4 Hz, H-7), 7.56 (1H, dd, *J*_m,o_=2.4 Hz, 9.0 Hz, H-9), 7.38 (1H, d, *J*_o_=9.0 Hz, H-10), 5.70-5.64 (1H, m, H-3'), 5.40 (1H, d, *J*_2',3'_=11.7 Hz, H-2'), 2.88 (1H, dd, *J*_1,3_=3.6 Hz, *J*_1,2_=4.8 Hz, H-1), 2.68 (1H, dd, *J*_3,1_=3.6 Hz, *J*_3,2_=9.0 Hz, H-3), 2.52 (1H, dd, *J*_2,1_=4.8 Hz, *J*_2,3_=9.0 Hz, H-2), 2.84-2.82 (1H, m, H-4'), 2.28-2.20 (1H, m, H-4'), 2.11-1.54 (8H, m, H-5',6',7',8'). MS (EI): *m/z* 384 (M^+^, 100%). Anal. Calcd. for C_22_H_21_ClO_4_: C, 68.66; H, 5.50. Found: C, 68.62; H, 5.48.

Compound **7a** (R = CH_3_)**.** Yield 18%, white solid; mp 170-172 ^o^C; *ν*_max_ (cm^-1^) 1605 (C=C), 1640 (C=O), 1694 (C=O); δ_H_ (CDCl_3_) 8.17 (1H, d, *J*_m_ 2.4 Hz H-7), 7.56 (1H, dd, *J*_m,o_=2.4 Hz, 9.0 Hz, H-9), 7.39 (1H, d, *J*_o_=9.0 Hz, H-10), 6.10-6.08 (1H, m, H-3'), 5.71-5.69 (1H, m, H-2'), 2.90 (1H, dd, *J*_1,3_=3.6 Hz, *J*_1,2_=4.8 Hz, H-1), 2.70 (1H, dd, *J*_3,1_=3.6 Hz, *J*_3,2_=9.0 Hz, H-3), 2.47 (1H, dd, *J*_2,1_=4.8 Hz, *J*_2,3_=9.0 Hz, H-2), 2.41 (3H, s, -COCH_3_), 2.38-2.21 (4H, m, H-4',5'). MS (EI): *m/z* 342 (M^+^, 100%). Anal. Calcd. for C_19_H_15_ClO_4_: C, 66.58 ; H, 4.41. Found: C, 66.62; H, 4.38.

Compound **7b** (R = CH_3_)**.** Yield 20%, white solid; mp 182-184 ^o^C; *ν*_max_ (cm^-1^) 1603 (C=C), 1641 (C=O), 1696 (C=O); δ_H_ (CDCl_3_) 8.19 (1H, d, *J*_m_=2.4 Hz, H-7), 7.58 (1H, dd, *J*_m,o_=2.4 Hz, 9.0 Hz, H-9), 7.41 (1H, d, *J*_o_=9.0 Hz, H-10), 6.12-6.10 (1H, m, H-3'), 5.73-5.70 (1H, m, H-2'), 2.96 (1H, dd, *J*_1,3_=3.6 Hz, *J*_1,2_=4.8 Hz, H-1), 2.78 (1H, dd, *J*_3,1_=3.6 Hz, *J*_3,2_=9.0 Hz, H-3), 2.51 (1H, dd, *J*_2,1_=4.8 Hz, *J*_2,3_=9.0 Hz, H-2), 2.45 (3H, s, -COCH_3_), 2.39-2.18 (6H, m, H-4',5',6'). MS (EI): *m/z* 356 (M^+^, 100%). Anal. Calcd. for C_20_H_17_ClO_4_: C, 67.32; H, 4.80. Found: C,67.28; H, 4.81.

Compound **7c** (R = CH_3_)**.** Yield 20%, white solid; mp 193-194 ^o^C; *ν*_max_ (cm^-1^) 1602 (C=C), 1658 (C=O), 1703 (C=O); δ_H_ (CDCl_3_) 8.15 (1H, d, *J*_m_=2.4 Hz, H-7), 7.54 (1H, dd, *J*_m,o_=2.4 Hz, 9.0 Hz, H-9), 7.38 (1H, d, *J*_o_=9.0 Hz, H-10), 5.68-5.62 (1H, m, H-3'), 5.46 (1H, d, *J*_2',3'_=11.4 Hz, H-2'), 2.92 (1H, dd, *J*_1,3_=3.6 Hz, *J*_1,2_=4.8 Hz, H-1), 2.68 (1H, dd, *J*_3,1_=3.6 Hz, *J*_3,2_=9.0 Hz, H-3), 2.50 (1H, dd, *J*_2,1_=4.8 Hz, *J*_2,3_=9.0 Hz, H-2), 2.37 (3H, s, -COCH_3_), 2.84-2.80 (1H, m, H-4'), 2.28-2.20 (1H, m, H-4'), 2.07-1.52 (8H, m, H-5',6',7',8'). MS (EI): *m/z* 384 (M^+^, 100%). Anal. Calcd. for C_22_H_21_ClO_4_: C, 68.66; H, 5.50. Found: C, 68.54; H, 5.55.
